# Administration of 3,5-diiodothyronine (3,5-T2) causes central hypothyroidism and stimulates thyroid-sensitive tissues

**DOI:** 10.1530/JOE-13-0502

**Published:** 2014-06

**Authors:** Alvaro Souto Padron, Ruy Andrade Louzada Neto, Thiago Urgal Pantaleão, Maria Carolina de Souza dos Santos, Renata Lopes Araujo, Bruno Moulin de Andrade, Monique da Silva Leandro, João Pedro Saar Werneck de Castro, Andrea Claudia Freitas Ferreira, Denise Pires de Carvalho

**Affiliations:** 1 Laboratório de Fisiologia Endócrina Doris Rosenthal Instituto de Biofísica Carlos Chagas Filho and Instituto de Pesquisa Translacional em Saúde e Ambiente na Região Amazônica (INPeTAM), CCS-Bloco G- Cidade Universitria Ilha do Fundo, Rio de Janeiro, 21949-900 Brazil; 2 Laboratório de Biologia do Exercício, Escola de Educação Física e Desportos Universidade Federal do Rio de Janeiro Rio de Janeiro Brazil

**Keywords:** 3,5-T2, TSH, thyroid hormone metabolism, deiodinase

## Abstract

In general, 3,5-diiodothyronine (3,5-T2) increases the resting metabolic rate and oxygen consumption, exerting short-term beneficial metabolic effects on rats subjected to a high-fat diet. Our aim was to evaluate the effects of chronic 3,5-T2 administration on the hypothalamus–pituitary–thyroid axis, body mass gain, adipose tissue mass, and body oxygen consumption in Wistar rats from 3 to 6 months of age. The rats were treated daily with 3,5-T2 (25, 50, or 75 μg/100 g body weight, s.c.) for 90 days between the ages of 3 and 6 months. The administration of 3,5-T2 suppressed thyroid function, reducing not only thyroid iodide uptake but also thyroperoxidase, NADPH oxidase 4 (NOX4), and thyroid type 1 iodothyronine deiodinase (D1 (DIO1)) activities and expression levels, whereas the expression of the TSH receptor and dual oxidase (DUOX) were increased. Serum TSH, 3,3′,5-triiodothyronine, and thyroxine were reduced in a 3,5-T2 dose-dependent manner, whereas oxygen consumption increased in these animals, indicating the direct action of 3,5-T2 on this physiological variable. Type 2 deiodinase activity increased in both the hypothalamus and the pituitary, and D1 activities in the liver and kidney were also increased in groups treated with 3,5-T2. Moreover, after 3 months of 3,5-T2 administration, body mass and retroperitoneal fat pad mass were significantly reduced, whereas the heart rate and mass were unchanged. Thus, 3,5-T2 acts as a direct stimulator of energy expenditure and reduces body mass gain; however, TSH suppression may develop secondary to 3,5-T2 administration.

## Introduction

Compound 3,3′,5-triiodothyronine (T_3_) exerts many important effects on the basal metabolic rate and increases oxygen consumption. Several years ago, it was shown that 3,5-diiodothyronine (3,5-T2) is responsible for certain non-genomic effects of thyroid hormones on the resting metabolic rate (RMR) and oxygen consumption (Lanni *et al*. 1996, Moreno *et al*. 2002). Compound 3,5-T2 by itself increases the rate of oxygen consumption in perfused rat liver (Horst *et al*. 1989), blood mononuclear cells (Kvetny 1992), and rat liver mitochondria (Lanni *et al*. 1992, 1993, O'Reilly & Murphy 1992) and affects the activity of enzymes involved in energy metabolism, including α-glycerophosphate dehydrogenase (Lombardi *et al*. 2000), cytochrome oxidase (Lanni *et al*. 1992, 1993, Goglia *et al*. 1994, Arnold *et al*. 1998), malate dehydrogenase (Ball *et al*. 1997, Lombardi *et al*. 2000), glucose-6-phosphate dehydrogenase (Lombardi *et al*. 2000), and F0F1-ATP synthase (Mangiullo *et al*. 2010). More recently, it has been shown that the administration of 3,5-T2 to rats fed on a high-fat diet leads to a liver proteome profile similar to that found in non-steatotic livers, which is likely due to the hypolipidemic effect of this thyroid hormone metabolite (Silvestri *et al*. 2010). Because Wistar rats spontaneously develop obesity as they age (Newby *et al*. 1990), chronic 3,5-T2 administration could be beneficial. In these previous studies, pharmacological doses of 3,5-T2 were administered, as Engler & Burger (1984) reported 3,5-T2 physiological serum concentrations to be around 1 ng/dl in men, with a daily production rate estimated to be around 1 μg (Engler & Burger 1984, Engler *et al*. 1984).

Apart from these unequivocal effects on energy metabolism, 3,5-T2 might also affect the hypothalamus–pituitary–thyroid axis. Horst *et al*. (1995) have shown that 3,5-T2 treatment reduced serum thyroid-stimulating hormone (TSH) and thyroxine (T_4_) levels as well as TSH secretion from rat pituitary fragments, and it was also able to decrease the TSH levels of hypothyroid rats (Moreno *et al*. 1998). Moreover, 3,5-T2 increases pituitary type 1 iodothyronine deiodinase (D1) activity and transiently decreases type 2 iodothyronine deiodinase (D2) activity in rats (Baur *et al*. 1997), while in killifish, short-term exposure to 3,5-T2 was shown to decrease D2 activity and *d2* (*dio2*) mRNA levels in the liver (García-G *et al*. 2007).

Despite the possible interference of 3,5-T2 on thyroid function (Horst *et al*. 1995, Baur *et al*. 1997, Moreno *et al*. 1998, García-G *et al*. 2007), to our knowledge, the chronic effects of 3,5-T2 on proteins involved in thyroid hormone biosynthesis (Kopp 2013) and thyroid hormone metabolism have not been assessed thus far. Therefore, our aim was to study the effect of chronic pharmacological 3,5-T2 administration on these parameters. Our goal is based on the fact that 3,5-T2 has been suggested to be a possible therapeutic tool to overcome liver steatosis and insulin resistance; hence, it is tempting to speculate as to whether its chronic administration could lead to thyroid dysfunction, thus affecting other organs that do not respond to 3,5-T2.

In this study, we describe the effects of chronic 3,5-T2 administration on body mass gain and the hypothalamus–pituitary–thyroid axis during aging in male Wistar rats. We show herein that chronic 3,5-T2 administration impairs aging-related body mass gain while suppressing TSH and normal thyroid function.

## Materials and methods

### Animals

Adult male Wistar rats were housed at a controlled temperature (23 °C) with daily exposure to a 12 h light:12 h darkness cycle and were provided free access to water and standard rat chow. This investigation conforms to the Guidelines for the Care and Use of Laboratory Animals published by the US National Institutes of Health (NIH Publication No.85-23, revised 1996) and was approved (number: IBCCF 070) by the Institutional Animal Care and Use Committee (Comissão de Ética no Uso de Animais do Centro de Ciências da Saúde, CEUA/CCS). All animals were individually housed for a 1-week acclimation period.

### Treatment with 3,5-T2

Three-month-old male Wistar rats were divided into the following groups: control (daily s.c. injections of vehicle were administered), 25 μg 3,5-T2 (daily s.c. injections of 25 μg 3,5-T2/100 g body weight (BW)), 50 μg 3,5-T2 (daily s.c. injections of 50 μg 3,5-T2/100 g BW), and 75 μg 3,5-T2 (daily s.c. injections of 75 μg 3,5-T2/100 g BW). The experiments were conducted for 3 months. Compound 3,5-T2 (Sigma) was dissolved in 0.04 M NaOH and subsequently diluted with saline solution (0.04 M NaOH and saline were the vehicle) at the time of administration (0.09% NaCl, 60% v/v; stock solution of 1 μg 3,5-T2/μl), which maintained equal alkalinity in all of the injected solutions. Animals were weighed at the beginning of the treatment and just before killing; food ingestion was determined for 24 h before killing. After the experimental period, animals were killed by decapitation, and blood was collected. The serum was obtained and stored at −20 °C until specific RIA to determine serum TSH, T_3_, and T_4_ could be carried out. Hearts, retroperitoneal fat pads (adipose tissue from the perirenal capsules and attached to the dorsal rat body wall, without the adrenal glands that are embedded in the perirenal adipose tissue), and epididymal fat pads were removed and weighed. Liver, kidneys, thyroid glands, hypothalami, and pituitaries were dissected out and stored at −70 °C until processing for enzymatic measurements or for RNA extraction procedure.

### Serum insulin, TSH, T_3_, and T_4_

The fasting serum insulin concentrations were measured using a commercial RIA kit for rat insulin (MP Biomedicals, LLC, Santa Clara, CA, USA, sensitivity of 5.5 μlU/ml), and inter- and intra-assay coefficient of variation (CV) values were 5.0 and 8.9% respectively.

The serum TSH levels were evaluated using a specific RIA obtained from the National Institute of Diabetes, Digestive and Kidney Diseases (NIDDK; Bethesda, MD, USA) and expressed in terms of the reference preparation 2 (RP2). The intra- and inter-assay CV values were 7.7 and 6.5% respectively and the sensitivity was 0.63 ng/ml.

Total serum T_3_ and T_4_ concentrations were measured using commercial RIA kits based on the presence of specific antibodies adhered to the internal surface of propylene tubes. T_3_: DLS – 3100 Active, sensitivity of 4.3 ng/dl, inter- and intra-assay CV values varied from 4.2 to 6.0% and from 5 to 6.5% respectively; T_4_: DLS – 3200 Active, sensitivity of 0.4 μg/dl, inter- and intra-assay CV values varied from 7.1 to 7.4% and from 2.9 to 5.1% respectively (DSL, TX, USA).

All procedures were carried out following the manufacturer's recommendations.

### Glucose tolerance test

A glucose tolerance test was carried out before the commencement of 3,5-T2 treatment and after 1, 2, and 3 months of treatment, as described previously (Yagil *et al*. 2005). Briefly, animals were kept on fasting overnight and blood was obtained from the tip of the tail of fully awake, non-anesthetized animals. Blood glucose levels were determined using a glucose reagent strip and a standard automated glucometer (AccuChek Advantage II, Roche). After the fasting blood glucose level was determined, animals were loaded by gavage with 3.5 g glucose/kg BW, and blood glucose levels were measured 30, 60, 120, and 180 min after loading by gavage. The results are expressed as the area under the glycemic curve.

Because a dose of 50 μg 3,5-T2/100 g BW impaired body weight gain and mildly improved glucose tolerance without significantly affecting heart weight, while significantly reducing serum T_3_, T_4_, and TSH levels, we have chosen this daily dose to study the effect of 3,5-T2 administration on thyroid function, thyroid hormone metabolism, and oxygen consumption.

### Resting metabolic rate

We evaluated the RMR of rats that received 50 μg 3,5-T2 (daily injections of 50 μg 3,5-T2/100 g BW) and control rats, as described previously (Araujo *et al*. 2008). The RMR was measured using open-circuit indirect calorimetry for 24 h, after 90 days of treatment. Rats were individually placed in a respiration chamber (24×21.5×20 cm); and the airflow was maintained constant at 700 ml/min by a mass flow controller (LE 400; Panlab, Barcelona, Spain). Oxygen was measured using an O_2_ analyzer (LE 405; Panlab). Spontaneous activity was measured in the same metabolic cage that contains four sensors in the housing base to detect the movement of the animal. Oxygen consumption and spontaneous activity were recorded at 5-min intervals, and the results were expressed in ml/min per kg^0.75^ of O_2_ and horizontal activity in counts/min respectively. To avoid disruption caused by adaptation to the chamber, the first 3 h (from 1800 to 2100) were excluded from the analyses.

### Electrocardiography

Electrocardiogram recording was carried out in conscious animals by the non-invasive method. Electrodes were positioned in specific positions and connected by flexible cables to a differential AC amplifier (model 1700, A-M Systems, Sequim, WA, USA), with a low pass signal filtered at 500 Hz, and digitalized at 1 kHz by a 16-bit A/D converter (AD Instruments, Colorado Springs, CO, USA) using the Lab chart 7.0 software (AD Instruments).

### Type 1 and type 2 iodothyronine deiodinase activities

The 5′-deiodinase activities in the liver, kidney, thyroid, pituitary, and hypothalamus were evaluated as described previously (Berry *et al*. 1991, Araujo *et al*. 2008, Fortunato *et al*. 2008). Briefly, 25 mg tissue samples (liver or kidney) or the whole thyroid, pituitary, and hypothalamus were homogenized in 1 ml of 0.1 M sodium phosphate buffer containing 1 mM EDTA (Merck), 0.25 M sucrose (Merck), and 10 mM dithiothreitol (DTT) (USB Products, Cleveland, OH, US) (pH 6.9). For the measurement of type 1 deiodinase activity, duplicate homogenates (30 μg protein from the liver or kidney and 15 μg protein from the thyroid) were incubated in a water bath for 1 h at 37 °C with 1 μM rT_3_ (Sigma), equal volumes of [^125^I] rT_3_ (PerkinElmer Life Sciences, Boston, MA, USA) previously purified using Sephadex LH-20, and 10 mM DTT (USB) in 100 mM potassium phosphate buffer containing 1 mM EDTA (pH 6.9). The reaction volume was 300 μl, in the presence or absence of propylthiouracil (PTU) (1 mM) in order to measure specific D1 activity. For the type 2 deiodinase activity assay, duplicate homogenates (30 μg protein from the hypothalamus and pituitary) were incubated in a water bath for 3 h at 37 °C with 1 nM T_4_ (Sigma), equal volumes of [^125^I] T_4_ (PerkinElmer Life Sciences) previously purified using Sephadex LH-20, 1 mM PTU, and 20 mM DTT (USB) in 100 mM potassium phosphate buffer containing 1 mM EDTA (pH 6.9). The reaction volume was 300 μl, in the presence or absence of excess of T_4_ (100 nM) in order to inhibit D2 in the blank assay tube. For D1, blank incubations were carried out in the absence of protein and the presence of 1 mM PTU. For D2, blank incubations were carried out in the presence of 100 nM T_4_.

After incubation, the reaction was terminated at 4 °C followed by the addition of 100 μl fetal bovine serum (Cultilab, Campinas, São Paulo, Brazil) and 200 μl trichloroacetic acid (50%, v/v). Samples were centrifuged at 8000 ***g*** for 3 min, and the supernatant was collected for the measurement of ^125^I liberated during the deiodination reaction. The protein concentration in the homogenates was measured by the Bradford method (Bradford 1976) after incubation of the homogenates with NaOH (2.5 M).

### Thyroid iodide uptake

We had previously demonstrated that the measurement of radioiodide uptake 15 min after ^125^I-NaI administration (short-term iodide uptake) reflects iodide transport through the sodium–iodide symporter (NIS) without the influence of *in vivo* thyroid iodine organification activity (Ferreira *et al*. 2005). Thus, to evaluate the NIS function *in vivo* using thyroid radioiodide uptake measurements, animals received Na-^125^I (3700 Bq, i.p.; Amersham) 15 min before decapitation. The thyroid glands were removed and weighed. The radioactivity of the thyroid glands was measured using a gamma counter (LKB) and expressed as percentage of total ^125^I injected per milligram of thyroid.

### Thyroperoxidase preparation and activity

Thyroperoxidase (TPO) extraction and activity measurement were carried out as described previously (Moura *et al*. 1989, Carvalho *et al*. 1994, Ferreira *et al*. 2005). Rat thyroids were minced and homogenized in 0.5 ml of 50 mM Tris–HCl buffer (pH 7.2) containing 1 mM KI using an Ultra-Turrax Homogenizer (IKA, Staufen, Germany). The homogenate was centrifuged at 100 000 ***g*** at 4 °C for 1 h. The pellet was suspended in 0.5 ml Triton (0.1%, v/v) and incubated at 4 °C for 24 h to solubilize TPO. The triton-treated suspension was centrifuged at 100 000 ***g*** at 4 °C for 1 h, and the supernatant containing solubilized TPO was used for the assays. The protein content was determined by the Bradford method (Bradford 1976).

To measure TPO iodide oxidation activity, assay mixtures were prepared containing 1.0 ml freshly prepared 50 mM sodium phosphate buffer (pH 7.4), 24 mM KI, 11 mM glucose, and increasing amounts of solubilized TPO. The final volume was adjusted to 2.0 ml with 50 mM sodium phosphate buffer (pH 7.4), and the reaction was initiated by the addition of 10 μl of 0.1% glucose oxidase (Boehringer Grade I). The increase in absorbance at 353 nm (triiodide production) was recorded for 4 min on a Hitachi spectrophotometer (U-3300). The ΔA353 nm/min was determined from the linear portion of the reaction curve and related to protein concentration. One unit of activity corresponds to ΔA353 nm/min=1.0.

### Thyroid H_2_O_2_ production

H_2_O_2_ generation was quantified in thyroid particulate fractions by the Amplex red/HRP assay (Molecular Probes, Invitrogen), which detects the accumulation of a fluorescent oxidized product. For particulate preparation, the excised thyroid glands remained at 4 °C for 24 h in 50 mM sodium phosphate buffer (pH 7.2) containing 0.25 M sucrose, 0.5 mM DTT, 1 mM EGTA, 5 mg/ml aprotinin, and 34.8 mg/ml phenylmethylsulfonyl fluoride (PMSF) before homogenization. Then, the homogenate was centrifuged at 100 000 ***g*** for 35 min at 4 °C and suspended in 0.25 ml of 50 mM sodium phosphate buffer (pH 7.2) containing 0.25 M sucrose, 2 mM MgCl_2_, 5 mg/ml aprotinin, and 34.8 mg/ml PMSF. This particulate fraction was incubated in 150 mM sodium phosphate buffer (pH 7.4) containing 100 U/ml superoxide dismutase (Sigma), 0.5 U/ml HRP (Roche), 50 μM Amplex red (Molecular Probes, Eugene, OR, USA), 1 mM EGTA, with or without 1.5 mM CaCl_2_, and the fluorescence was immediately measured in a microplate reader (Victor X4; PerkinElmer, Norwalk, CT, USA) at 30 °C, using wavelength excitation at 530 nm and emission at 595 nm. The H_2_O_2_ production was quantified using standard calibration curves.

### Immunoblotting of NIS, TSHR, dual oxidase, and NADPH oxidase 4

Thyroids were placed in lysis buffer containing 135 mM NaCl, 1 mM MgCl_2_, 2.7 mM KCl, 20 mM Tris (pH 8.0), 1% Triton, 10% glycerol, and protease and phosphatase inhibitors (0.5 mM Na_3_VO_4_, 10 mM NaF, 1 M leupeptin, 1 M pepstatin, 1 M okadaic acid, and 0.2 mM PMSF) and subsequently homogenized using an Ultra-Turrax Homogenizer (IKA). The samples were then centrifuged at 570 ***g*** for 10 min at 4 °C, and the supernatant was collected. An aliquot was used to determine the protein concentration via the BCA protein assay kit (Pierce, Rockford, IL, USA, catalog no. 23 227), following the manufacturer's instructions. The protein samples were then resolved by SDS–PAGE, transferred onto PVDF membranes, and probed with the indicated antibodies. The NIS antibody was kindly provided by Dr Nancy Carrasco, the dual oxidase (DUOX) antibody was kindly provided by Dr Corinne Dupuy, the anti-NADPH oxidase 4 (NOX4) primary antibody (NB 110-58851) was obtained from Novus Biologicals (Littleton, CO, USA), and TSHR antibody was purchased from Santa Cruz Biotechnology. GAPDH was used as an internal control (the antibody was purchased from Millipore Corporation, Billerica, WA, USA), and an anti-rabbit IgG HRP-linked antibody (Cell Signaling Technology, Boston, MA, USA) was used as a secondary antibody. The immunoblots were developed using ECL.

### Real-time PCR

Thyroid and hypothalamic total RNAs were extracted using the RNeasy Plus Mini Kit (Qiagen) following the manufacturer's instructions. After DNase treatment, RT was carried out followed by real-time PCR as described previously (Schmittgen *et al*. 2008). Specific oligonucleotides, as described in Supplementary Table 1, see section on supplementary data given at the end of this article, were purchased from Applied Biosystems. For each gene tested, the amplification of different cDNA quantities was linear in the PCR assay conditions used. In this experiment, β-actin was used as an internal control, and the results are expressed as ΔΔ*C*T.

### Statistical analyses

The results are expressed as the mean±s.e.m. Body, heart, and fat mass data were analyzed by one-way ANOVA followed by Dunnett's multiple comparison test. Serum T_4_ and T_3_ were analyzed by ANOVA followed by the Bonferroni multiple comparison test. Areas under the glycemic curve data were analyzed by two-way ANOVA followed by the Bonferroni post-test. The serum TSH results were analyzed using the non-parametric Kruskal–Wallis test followed by Dunn's multiple comparison test. The area under the curve data for oxygen consumption, food intake, thyroid iodide uptake, TPO activity, deiodinase activities, NIS, and TSHR protein levels as well as *Nis* (*Slc5a5*), *Tpo*, *D1* (Dio1) and *Tshr* mRNA levels were analyzed by the unpaired *t*-test. Statistical analyses were carried out using the GraphPad Prism software (version 4; GraphPad Software, Inc., San Diego, CA, USA). Differences were considered to be statistically significant when *P*<0.05.

## Results

### Body, heart, and fat mass

**Figure 1:**
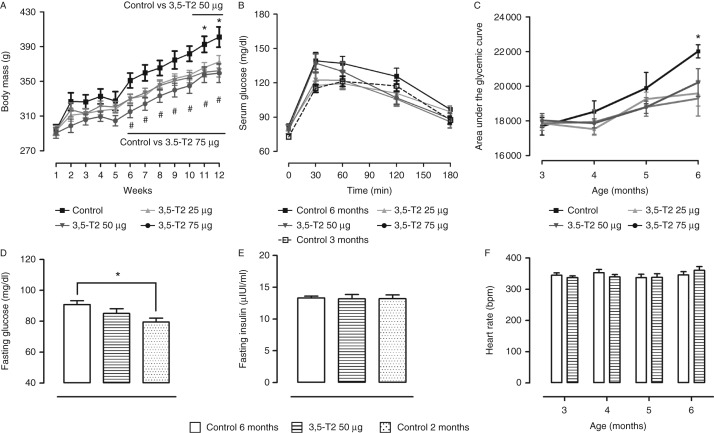
Male Wistar rats were treated (or not treated) with 3,5-diiodothyronine (3,5-T2) at doses of 25, 50, and 75 μg/100 g BW for 90 days. (A) Body mass during 90 days, *n*=14–16 animals per group; *P<0.05 control vs 50 μg 3,5-T2/100 g BW, ^#^ *P*<0.05 control vs 75 μg 3,5-T2/100 g BW. (B) Serum glucose after 90 days of treatment, five to nine animals per group; *P<0.05 control vs other groups. (C) Area under the glycemic curve obtained via glucose tolerance testing at different time points, five to nine animals per group. The test was carried out at the beginning of the treatment with 3,5-T2 (3 months of age) and 1, 2, and 3 months later. (D) Fasting serum glucose, 12–15 animals per group; *P<0.05 control (6 months) vs control (2 months). (E) Fasting serum insulin, six to eight animals per group. (F) Heart rate, 10–11 animals per group. The results are expressed as the mean±s.e.m. of the area under the glycemic curve. The data were analyzed by two-way ANOVA followed by the Bonferroni multiple comparison test.

After 3 months of 3,5-T2 administration, the final body mass was significantly reduced when compared with the control group independent of the dose (25, 50, or 75 μg of 3,5-T2/100 g BW). Body mass gain during the treatment was ≈30% lower in rats treated with 3,5-T2 when compared with those in the control group (Fig. 1A). Absolute heart mass did not differ among the groups, whereas relative heart mass was significantly increased in rats treated with the highest dose (75 μg) of 3,5-T2/100 g BW, which was mainly due to decreased body mass rather than increased heart mass (Table 1). Heart rate did not differ among the groups (Fig. 1F), as described previously (Lanni *et al*. 2005). Moreover, although retroperitoneal fat pad mass was significantly reduced in rats treated with 3,5-T2 independent of the 3,5-T2 dose used, the epididymal fat mass remained unchanged (Table 2).

### Glucose tolerance test

Control male Wistar rats developed impaired glucose tolerance at 6 months of age (Fig. 1B), and basal fasting serum glucose was significantly higher in the 6-month-old animals when compared with 3,5-T2-treated 6-month-old rats and control 2-month-old rats (Fig. 1D). Treatment with 3,5-T2 improved glucose tolerance at all doses (as shown in Fig. 1B). Animals treated with 3,5-T2 for 3 months have areas under the glycemic curve that are ∼10% lower than their paired controls (6 months old) (Fig. 1C). The change in the glucose tolerance noted in control aged rats may be related to the higher body mass observed in this group, which was significantly prevented by 3,5-T2 administration to rats. It is noteworthy that serum fasting insulin levels did not differ among the groups (Fig. 1E).

### Serum TSH, T_3_, and T_4_

**Figure 2:**
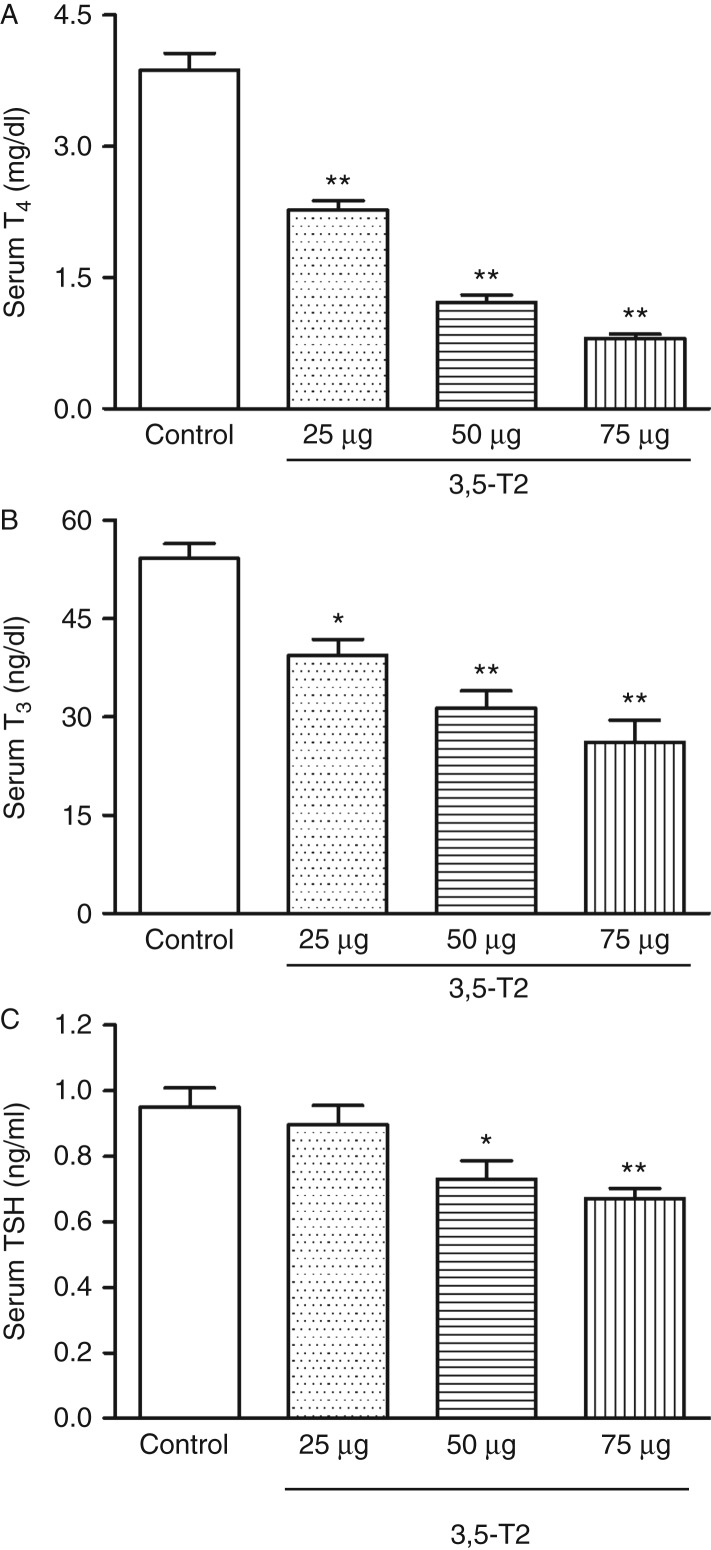
Serum total T_4_, T_3_, and TSH concentrations in rats treated (or not treated) with 3,5-diiodothyronine (3,5-T2) for 90 days at doses of 25, 50, and 75 μg/100 g BW. Hormone concentrations were determined using specific RIA. (A) Serum total T_4_ (13–18 rats per group); (B) serum total T_3_ (13–18 rats per group); and (C) serum TSH (11–14 rats per group). The results are expressed as the mean±s.e.m. Serum total T_4_ and T_3_ were analyzed by one-way ANOVA followed by the Bonferroni multiple comparison test, and serum TSH was analyzed by the non-parametric Kruskal–Wallis test followed by Dunn's multiple comparison test. *P<0.01 vs control group; **P<0.001 vs control group.

Serum total T_3_ and T_4_ levels were reduced in rats treated with 3,5-T2, and the reduction was dose dependent (Fig. 2A and B). Despite the decreased serum thyroid hormones, TSH levels remained normal in rats receiving 25 μg of 3,5-T2/100 g BW and were significantly lower in rats that received 50 and 75 μg of 3,5-T2/100 g BW (Fig. 2C), demonstrating that 3,5-T2 by itself might be exerting the negative feedback, as suggested previously (Horst *et al*. 1995, Moreno *et al*. 1998).

### Resting metabolic rate

**Figure 3:**
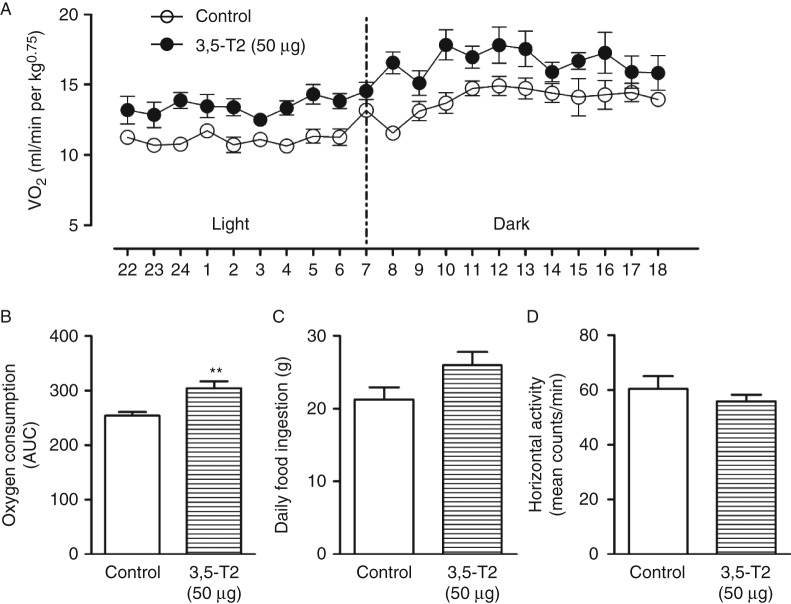
Resting metabolic rate (RMR) and food intake of control rats and rats treated with 3,5-diiodothyronine (3,5-T2) for 90 days at a dose of 50 μg/100 g BW. The RMR was measured using open-circuit indirect calorimetry for 21 h after 90 days of treatment (five rats per group). (A) Oxygen consumption; (B) area under the curve (AUC) of oxygen consumption (five rats per group); (C) 24 h food intake (nine rats per group); and (D) spontaneous daily activity as counts/min (eight rats per group). The results are expressed as the mean±s.e.m. The AUC of oxygen consumption and 24 h food intake were analyzed by the unpaired *t*-test. **P<0.001 vs control group.

Figure 3A summarizes the oxygen consumption results collected over a 21-h period of time, from 1800 1 day to 2200 the following day. The results are also expressed as the area under the curve of oxygen consumption for 21 h (Fig. 3B). Although 3,5-T2-treated rats had significantly decreased levels of serum T_3_ (Fig. 2B), which is the main known regulator of the RMR, oxygen consumption was increased in these animals. Food intake did not significantly differ between the groups (Fig. 3C). Our results reinforce previous data showing that 3,5-T2 can increase the RMR in rats.

### Type 1 iodothyronine deiodinase activity

**Figure 4:**
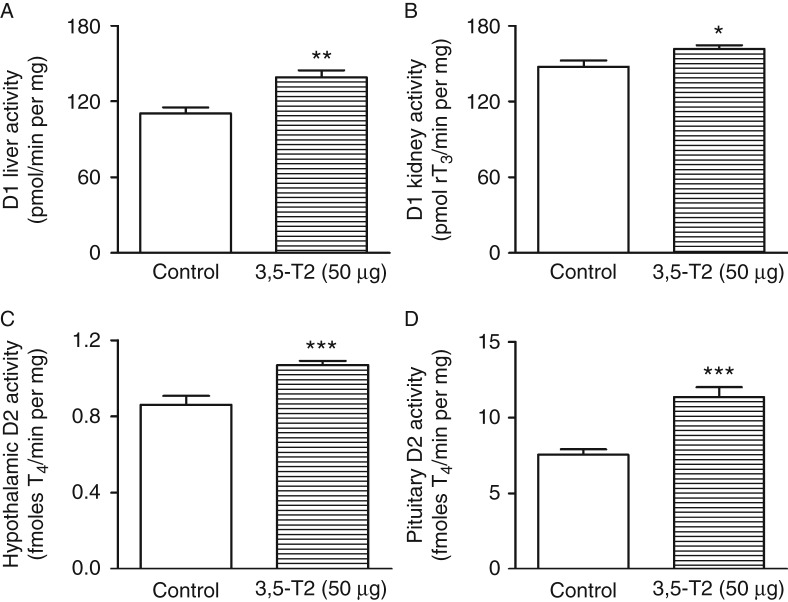
Type 1 (D1) and type 2 (D2) iodothyronine deiodinase activities in control rats and rats treated with 3,5-diiodothyronine (3,5-T2) for 90 days at a dose of 50 μg/100 g BW. (A) Hepatic D1 (control, *n*=12; 3,5-T2, *n*=17); (B) kidney D1 (control, *n*=8; 3,5-T2, *n*=12); (C) hypothalamic D2 (control, *n*=8; 3,5-T2, *n*=11); and (D) pituitary D2 (control, *n*=15; 3,5-T2, *n*=20) activities. The results are expressed as the mean±s.e.m. The data were analyzed by the unpaired *t*-test. *P<0.05 vs control group; **P<0.005 vs control group; ***P<0.0005 vs control group.

Liver and kidney D1 activities were significantly higher in rats treated with 3,5-T2 (Fig. 4A and B), even in the presence of significantly lower serum T_3_, which is the main stimulator of hepatic D1 expression. This result indicates that 3,5-T2 has a direct stimulatory effect on both kidney and hepatic D1 activities, again simulating the well-described genomic action of T_3_ (Araujo *et al*. 2008). These results, together with the data on TSH regulation, strongly support the hypothesis that 3,5-T2 functions as an agonist of the β isoform of thyroid hormone receptor (TR) *in vivo*.

### Type 2 iodothyronine deiodinase activity

Treatment with 3,5-T2 is associated with a significant increase in both hypothalamic and pituitary D2 activities (Fig. 4C and D). Presumably, the increase in D2 could be related to the decreased serum T_4_ levels and not to 3,5-T2 treatment itself. It is tempting to speculate that the increased hypothalamic and pituitary D2 activities might lead to an increase in local T_3_ production and thus could result in inappropriately normal or even decreased TSH in the presence of low serum T_4_.

### NIS, TPO, DUOX1, DUOX2, NOX4, D1, TSHR, and TRH expressions

**Figure 5:**
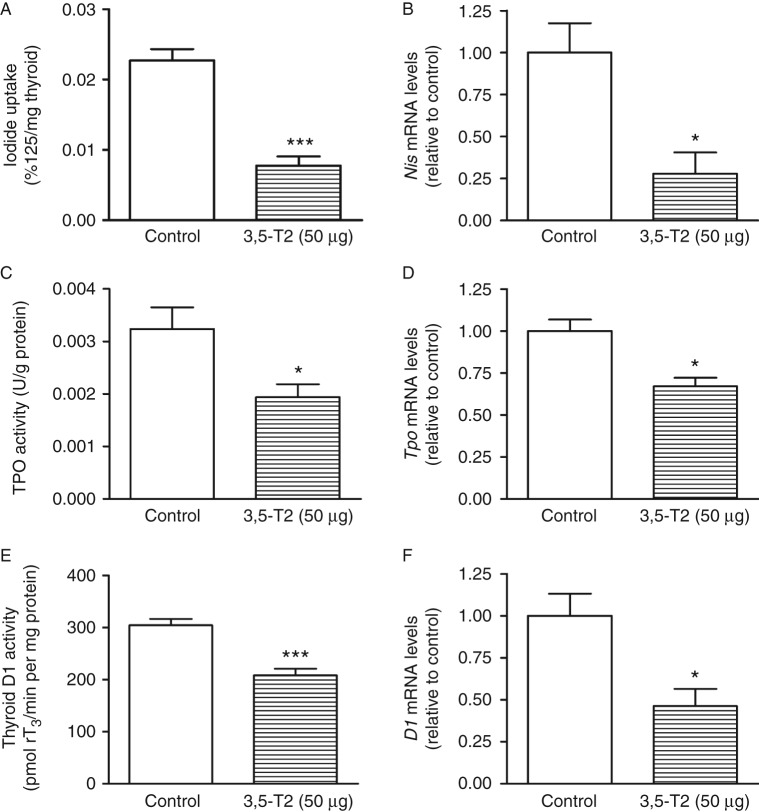
Thyroid iodide uptake, *Nis* mRNA levels, thyroperoxidase (TPO) activity, *Tpo* mRNA levels, thyroid type 1 iodothyronine deiodinase (D1) activity, and thyroid *D1* mRNA levels in control rats and rats treated with 3,5-diiodothyronine (3,5-T2) for 90 days at a dose of 50 μg/100 g BW. (A) *In vivo* thyroid iodide uptake (control, *n*=11; 3,5-T2, *n*=11); (B) *Nis* mRNA levels (control, *n*=5; 3,5-T2, *n*=6); (C) *in vitro* TPO iodide oxidation activity (control, *n*=12; 3,5-T2, *n*=10); (D) *Tpo* mRNA levels (control, *n*=5; 3,5-T2, *n*=5); (E) *in vitro* thyroid D1 activity (control, *n*=7; 3,5-T2, *n*=10); and (F) thyroid *D1* mRNA levels (control, *n*=5; 3,5-T2, *n*=6). The results are expressed as the mean±s.e.m. The data were analyzed by the unpaired *t*-test. *P<0.05 vs control group; ***P<0.0005 vs control group.

**Figure 6:**
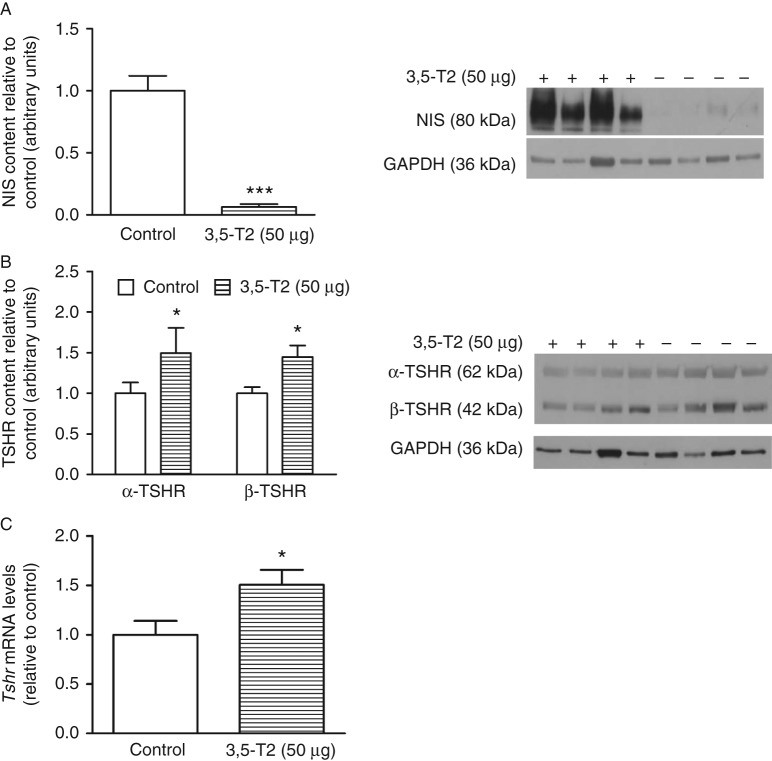
NIS and TSH receptor (TSHR) protein and *Tshr* mRNA levels in control rats and rats treated with 3,5-diiodothyronine (3,5-T2) for 90 days at a dose of 50 μg/100 g BW. Thyroid lysates were prepared, and western blot analysis was carried out with 30 μg protein using anti-NIS (control, *n*=11; 3,5-T2, *n*=11) (A) and anti-TSHR (control, *n*=10; 3,5-T2, *n*=10) (B) antibodies. GAPDH was used as an internal control. The NIS antibody was diluted 1:1000, the TSHR antibody was diluted 1:500, the GAPDH antibody was diluted 1:4000, and the secondary antibody was diluted 1:1500. (C) Thyroid *Tshr* mRNA levels (control, *n*=5; 3,5-T2, *n*=4). Molecular weights of the proteins: NIS corresponds to 80 kDa, α-TSHR to 62 kDa, β-TSHR to 42 kDa, and GAPDH to 36 kDa. The results are expressed as the mean±s.e.m. The data were analyzed by the unpaired *t*-test. *P<0.05 vs control group; ***P<0.001 vs control group.

As shown in Fig. 5A, B and C, 3,5-T2 administration significantly reduced thyroid iodide uptake, TPO iodide oxidation activity, and thyroid D1 activity together with the significantly lower *Nis*, *Tpo*, and *D1* mRNA levels (Fig. 5B, D and F). These results are in accordance with the lower serum thyroid hormone levels and might be secondary to the decreased serum TSH levels. To evaluate whether the reduction in NIS function in 3,5-T2-treated rats was due to decreased NIS protein expression, we evaluated the thyroid NIS content. As shown in Fig. 6A, the NIS protein content was greatly reduced by 3,5-T2 treatment, in accordance with the decreased thyroid iodide uptake and reduced serum TSH.

As TSH is the main regulator of thyroid function, we also evaluated the effect of 3,5-T2 on TSH receptor (TSHR) protein levels. The protein level of both α- and β-TSHR subunits (Fig. 6B) and the *Tshr* mRNA content (Fig. 6C) were significantly higher in the thyroids of 3,5-T2-treated rats, which might at least partially counterbalance the reduced serum TSH levels.

Hypothalamic *Trh* mRNA content did not significantly differ between the groups (C=1.00±0.14; 3,5-T2=0.80±0.05, *n*=6).

**Figure 7:**
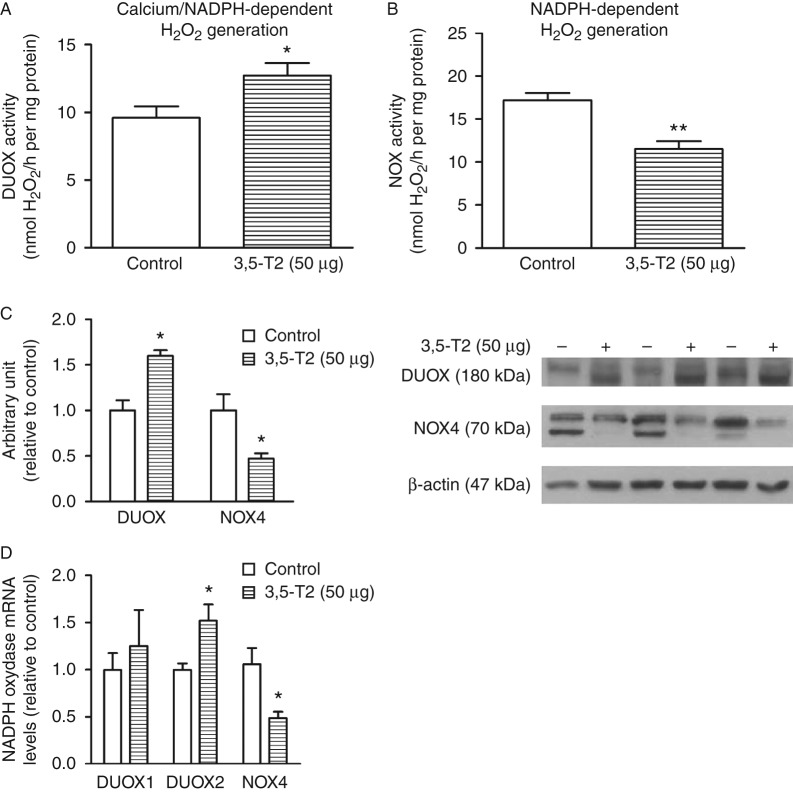
Thyroid calcium and NADPH-dependent hydrogen peroxide generation and DUOX and NOX4 protein levels in control rats and rats treated with 3,5-diiodothyronine (3,5-T2) for 90 days at a dose of 50 μg/100 g BW. (A) Calcium-dependent and (B) thyroid NADPH-dependent H_2_O_2_-generating activity was measured by the Amplex red method (control, *n*=8; 3,5-T2, *n*=8); (C) thyroid lysates were prepared, and western blot analysis was carried out with 30 μg protein using anti-DUOX (control, *n*=4; 3,5-T2, *n*=4) and anti-NOX4 (control, *n*=4; 3,5-T2, *n*=4) antibodies. GAPDH was used as an internal control. DUOX antibody was diluted 1:2000, NOX4 antibody was diluted 1:1000, β-actin antibody was diluted 1:5000, and the secondary antibody was diluted 1:4000. (D) Thyroid DUOX1, DUOX2 and NOX4 mRNA levels (control *n*=5; 3,5-T2 *n*=4). The results are expressed as mean±s.e.m. The data were analyzed by the unpaired *t*-test. *P<0.05 vs control group; **P<0.005 vs control group.

In addition, NOX4 that is positively regulated by TSH in the thyroid (Weyemi *et al*. 2010) was significantly decreased, as well as the calcium-independent H_2_O_2_ generation (Fig. 7B, C and D). By contrast, DUOX calcium-dependent H_2_O_2_ generation increased due to higher DUOX2 expression (Fig. 7A, C and D).

## Discussion

Recent data have demonstrated the beneficial effects of 3,5-T2 in preventing liver steatosis in animals fed on a high-fat diet (Silvestri *et al*. 2010), and these authors claimed that 3,5-T2 exerts non-genomic effects. These interesting results prompted us to evaluate whether the chronic administration of 3,5-T2 to aging Wistar rats could prevent the natural development of overweight that occurs in this rat strain. We were also interested in its possible undesirable effects on the pituitary–thyroid axis.

We show herein that daily 3,5-T2 administration to rats for a period of 90 days leads to a reduction in body mass gain and retroperitoneal fat mass, accompanied by a higher RMR despite the lower serum T_4_ and T_3_ levels. Regarding body composition, we did not analyze whether the treated animals lost lean body mass; however, because the metabolic rate is corrected by weight, a preservation of the lean body mass could explain the increase in the metabolic rate. Decreased body mass gain in 3,5-T2-treated rats was not dose dependent, suggesting that a maximal effect was already obtained with 25 μg of 3,5-T2/100 g BW; however, it is possible that the decrease in serum T_4_ and T_3_ levels might in some way compensate for the increase in the 3,5-T2 dose. Horst *et al*. (1995) did not observe any changes in body mass gain in rats treated for 3 months with the same dose of 3,5-T2. A possible explanation for the discrepancy between these results and ours is that these authors administered 3,5-T2 in the drinking water, which could reduce the bioavailability of the drug, whereas in this study, 3,5-T2 was injected subcutaneously. On the other hand, Lanni *et al*. (2005) showed that 25 μg of 3,5-T2/100 g BW were indeed able to reduce the body mass gain and adiposity of rats, whereas no changes in serum free T_4_ were detected in animals subjected to a high-fat diet and treated with 3,5-T2. It is important to note that in this previous report, 3,5-T2 was not administered to control animals (Lanni *et al*. 2005). Additionally, Lombardi *et al*. (2009) demonstrated that 3,5-T2 rapidly enhances the fatty acid oxidation rate in skeletal muscle. Apart from the chronic effects of 3,5-T2, acute and most likely non-genomic actions have also been described, such as increased rat liver mitochondrial respiratory rates (Lanni *et al*. 1993), hepatic oxygen consumption (Horst *et al*. 1995), respiration rate in the liver of rats fed on a high-fat diet (Mollica *et al*. 2009), and energy expenditure restoration in hypothyroid rats (Cimmino *et al*. 1996). Thus, these previously described metabolic effects of 3,5-T2 might explain the reduction in body mass gain and retroperitoneal adiposity observed in aged rats treated with 3,5-T2. The epididymal fat pad is important for reproductive function and has been shown to be necessary for the maintenance of spermatogenesis (Chu *et al*. 2010). Furthermore, the retroperitoneal fat pad appears to be more sensitive to thyroid hormone action than epididymal fat, as previously reported in hyperthyroid rats (Blennemann *et al*. 1992); therefore, it is unsurprising that 3,5-T2 treatment did not significantly affect epididymal fat mass in this study.

In addition to the beneficial effects of 3,5-T2 on body mass gain and adiposity, rats treated with 3,5-T2 showed an improvement in the glucose tolerance test by more than 10% when compared with control animals. This result suggests that 3,5-T2 mildly improves glucose tolerance, either directly or due to the decreased adiposity observed in the treated animals. To our knowledge, this is the first report describing the beneficial effect of chronic 3,5-T2 treatment on *in vivo* glucose homeostasis in 6-month-old Wistar rats. These results are in accordance with the finding that 3,5-T2 prevents insulin resistance in the skeletal muscle of rats fed on a high-fat diet and increases GLUT4 (SLC2A4) protein expression and insulin-induced Akt phosphorylation (Moreno *et al*. 2011). In addition to its other effects on the liver, 3,5-T2 also increases nuclear sirtuin 1 and downregulates lipogenic genes (De Lange *et al*. 2011).

In accordance with previous data showing that 3,5-T2 exerts important metabolic effects (Lanni *et al*. 1996, Lombardi *et al*. 2007, 2009), we have found a significant increase in the oxygen consumption of rats treated with 50 μg of 3,5-T2/100 g BW when compared with control animals. It is important to note that these animals had significantly lower serum T_3_ and T_4_ levels, which are the main regulators of energy metabolism. It has indeed been shown that 3,5-T2 acutely enhances mitochondrial fatty acid oxidation rates and thermogenesis in rat skeletal muscle (Lombardi *et al*. 2009), increases hepatic lipid β-oxidation (Lanni *et al*. 2005, Lombardi *et al*. 2009), and increases mitochondrial oxygen consumption (Mollica *et al*. 2009) in addition to increasing resting metabolism (Moreno *et al*. 2002). Thus, it is tempting to speculate that 3,5-T2 by itself functions as a chronic stimulator of the RMR, increasing oxygen consumption.

Regardless of the reduced serum T_4_ and T_3_ levels, TSH was lower in 3,5-T2-treated animals. However, Antonelli *et al*. (2011) reported no changes in serum thyroid hormone levels in two euthyroid subjects treated for 3 weeks with a daily dose of 300 μg of 3,5-T2 (Antonelli *et al*. 2011). Horst *et al*. (1995) showed that 3,5-T2 reduces TSH secretion by rat pituitary fragments stimulated by TRH. These authors also found a reduction in serum T_4_ levels after 90 days of treatment with 25 μg of 3,5-T2/100 g BW, which is in accordance with the results obtained herein. Moreover, a single dose of 3,5-T2 was shown to reduce serum *Tsh* and pituitary β-TSH (*Tshb*) mRNA levels (Baur *et al*. 1997). As reviewed by Williams & Bassett (2011), TRβ is the main mediator of thyroid hormone negative feedback. The findings of Ball *et al*. (1997), showing that TRβ2 binds to 3,5-T2 with greater affinity than the other TR isoforms, could well explain the effectiveness of 3,5-T2 for suppressing TSH secretion. Recently, it has also been shown that 3,5-T2 binds to and activates the human THRB isoform of TRs, again showing that this metabolite exerts genomic effects (Mendoza *et al*. 2013). Based on our results, we cannot explain as to why serum thyroid hormone levels were already decreased at a dose of 25 μg 3,5-T2/100 g BW, while serum TSH levels remained in the normal range. It is possible that despite the normal TSH concentration, the hormone could be less bioactive, which could lead to less stimulation of the thyroid and the consequent reduction in T_3_ and T_4_ production, although the hypothalamic content of *Trh* mRNA did not change in 3,5-T2-treated rats.

Hepatic and kidney D1 activities were significantly increased in rats treated with 3,5-T2 despite significantly reduced serum T_3_ levels. As T_3_ is the main stimulator of hepatic and kidney D1 activities (Koenig 2005, Gereben *et al*. 2008) through genomic actions, our results reinforce the idea of a T_3_-like genomic effect of 3,5-T2 on D1 regulation (rather than a non-genomic action). The regulation of the *D1* gene by T_3_ is mediated by the TRβ isoform because T_3_ induction of hepatic and renal D1 is severely weakened in *Thrb* *1* (*Thrb*)-null mice but is normal in *Thra* *1* (*Thra*)-null mice (Koenig 2005). Thus, 3,5-T2 might activate TRβ, leading to increased D1 activity in the liver and kidney once type 1 deiodinase is regulated at the transcriptional level. In accordance with our data, Baur *et al*. (1997) showed that 3,5-T2 stimulates D1 activity in rat anterior pituitaries *in vivo*. By contrast, in the thyroid gland, D1 activity is under the positive control of TSH through the cAMP pathway (St Germain & Galton 1997). As a result, the decreased serum TSH levels in rats receiving 3,5-T2 explain the reduction in their thyroid D1.

On the other hand, D2 activity was increased in both the hypothalamus and pituitary of rats treated with 3,5-T2, despite the thyromimetic effects of 3,5-T2. It has been shown that D2 protein levels and activity are regulated by post-translational mechanisms, especially ubiquitination (Gereben *et al*. 2000), which appears to be induced mainly by T_4_. As T_4_ levels are reduced in 3,5-T2-treated rats, this could explain the increase in D2 activity in these animals.

Taken together, our results show that 3,5-T2 significantly downregulates thyroid function. In addition to the decreased levels of thyroid hormones, we have also observed a reduction in the thyroid D1, NIS, and TPO activities as well as their respective mRNA levels. Iodide uptake is a fundamental step in thyroid hormone biosynthesis, and TPO is a key enzyme in thyroid hormone biosynthesis (Dai *et al*. 1996, Carvalho & Ferreira 2007); therefore, the decreased thyroid hormone levels appear to be secondary to the decreased NIS function and TPO activity. The inhibitory effect of 3,5-T2 on thyroid function is at least in part due to the decreased serum TSH levels because TSH is the main stimulator of the thyroid gland. TSHR protein and *Tshr* mRNA levels were increased in the thyroids of 3,5-T2-treated rats. It has been shown that TSH exerts inhibitory effects on *Tshr* promoter activity (Ikuyama *et al*. 1997). Thus, it is conceivable that thyroid TSHR expression was higher in rats treated with 3,5-T2 because the serum TSH levels were significantly reduced. In addition, NOX4 expression is downregulated, while DUOX2 is upregulated, just as previously demonstrated in other models of decreased TSH action on thyrocytes (Milenkovic *et al*. 2007, Weyemi *et al*. 2010).

In conclusion, chronic 3,5-T2 administration reduced body mass gain and retroperitoneal fat mass and increased the RMR regardless of decreased thyroid hormone levels. The reduction in thyroid hormone levels might be secondary to the decreased serum TSH levels, leading to the reduced activity and expression of NIS, thyroid D1, and TPO. These new data support the idea that exogenous 3,5-T2 causes TSH suppression. Thus, future studies on the possible deleterious effects of hypothyroidism in tissues where 3,5-T2 might not act as a thyromimetic agent are of great importance before this thyroid metabolite can be used as a pharmacological agent.

## Supplementary data

This is linked to the online version of the paper at http://dx.doi.org/10.1530/JOE-13-0502.

Supplementary Table

## Figures and Tables

**Table 1:** Body mass of 3-month-old rats at the beginning of a 3-month treatment with 3,5-T2 (25, 50, and 75 μg 3,5-T2/100 g BW, s.c.) and at the end of the treatment. Body mass gain during this period, heart mass, and relative heart mass at the end of the experiment were also recorded. The data are expressed as the mean±s.e.m. and were analyzed by one-way ANOVA followed by Dunnett's multiple comparison test. The study included 14–16 animals per group

**Group**	**Body mass** (g) (3 months old)	**Body mass** (g) (6 months old)	**Body mass gain** (g)	**Heart mass** (g)	**Relative heart mass** (g/100 g BW)
Control	291.6±5.91	401.0±11.68	105.7±6.82	1.15±0.030	0.29±0.009
25 μg 3,5-T2	293.3±5.22	372.6±7.23*	85.1±4.99*	1.08±0.034	0.30±0.009
50 μg 3,5-T2	294.2±5.12	369.0±9.11*	78.8±6.91*	1.16±0.031	0.32±0.010
75 μg 3,5-T2	292.0±5.59	359.4±10.87*	77.7±5.62*	1.17±0.038	0.33±0.012*

*P<0.05 vs control group.

**Table 2:** Retroperitoneal and epididymal fat pad mass of 6-month-old rats treated with different doses of 3,5-T2 for 3 months (25, 50, and 75 μg 3,5-T2/100 g BW, s.c.). The data are expressed as the mean±s.e.m. and were analyzed by one-way ANOVA followed by Dunnett's multiple comparison tests. The study included 14–15 animals per group

**Group**	**Retroperitoneal fat mass** (g)	**Retroperitoneal fat** (g/100 g BW)	**Epididymal fat mass** (g)	**Epididymal fat** (g/100 g BW)
Control	7.64±0.88	1.92±0.84	7.53±0.67	1.89±0.58
25 μg 3,5-T2	5.05±0.46*	1.52±0.40	6.12±0.58	1.68±0.48
50 μg 3,5-T2	5.67±0.51*	1.59±0.42	6.10±0.53	1.64±0.46
75 μg 3,5-T2	5.48±0.58*	1.60±0.44	6.45±0.54	1.72±0.56

*P<0.05 vs control.
