# Supplementary material for: Administration of 3,5-diiodothyronine (3,5-T2) causes central hypothyroidism and stimulates thyroid-sensitive tissues
Source: J Endocrinol. 2014 Apr 1;221(3):415–27. doi: 10.1530/JOE-13-0502 (PMC4045230; doi:10.1530/JOE-13-0502)
Supplement: Supplementary Table [file supp_221_3_415__index.html]

Administration of 3,5‐diiodothyronine (3,5‐T2) causes central hypothyroidism and stimulates thyroid‐sensitive tissues — Effect of 3,5-diiodothyronine on thyroid axis — Supplementary Table 

# Administration of 3,5‐diiodothyronine (3,5‐T2) causes central hypothyroidism and stimulates thyroid‐sensitive tissues

## Supplementary Table

**Files in this Data Supplement:**

- Supplemental Table 1 - (PDF 34 KB)
