# Supplementary material for: Administration of 3,5-diiodothyronine (3,5-T2) causes central hypothyroidism and stimulates thyroid-sensitive tissues
Source: J Endocrinol. 2014 Apr 1;221(3):415–27. doi: 10.1530/JOE-13-0502 (PMC4045230; doi:10.1530/JOE-13-0502)
Supplement: Supplementary Table [file supp_JOE-13-0502_Supplementary_table_1.pdf]

Supplemental Table 1- Oligonucleotide sequences used for qRT-PCR

**Genes**	***Forward***	***Reverse***
**β-actin**	5’ – CACTTTCTACAATGAGCTGCG - 3’	5’ – CTGGATGGCTACGTACATGG – 3’
**D1**	5’ – GTGATACAGGAAGGCAGGATC – 3’	5’ – GAACGAAGGTCAAAGCTAAAGG – 3’
**DUOX1**	5’ – ATT TCT TGG GAG GTA CAG CG – 3’	5’ – GTT AGG CAG GTA GGG TTC TTT C – 3’
**DUOX2**	5’ – TGC TCT CAA CCC CAA AGT G – 3’	5’ – TCT CAA ACC AGT AGC GAT CAC – 3’
**NIS**	5’ – GCTCATCCTGAACCAAGTGA – 3’	5’ – ACGAGCATTACCACAACCTG – 3’
**TPO**	5’ – GAA TGA GGA ACT GAC CGA GAG – 3’	5’ – TGA CAA GCC ACA GAA CTC TC – 3’
**TRH**	5'-GAA CGT CGA TTC TTG TGG AAA G-3'	5'-TTC TCC CAA GTC TCC CCT C-3'
**TSHR**	5’ – AGG TCC CTT GGA AAA ATG AGG – 3’	5’ – GTC TCG AGT AGC TTC AGA GTC – 3’

-actin (beta- actin), **D1** (type 1 deiodinase), **DUOX1** (Dual Oxidase 1), **DUOX2** (Dual Oxidase 2), **NIS** (sodium-iodide symporter), **TPO** (thyroperoxidase), **TRH** (thyrotrophic hormone), **TSHR** (thyrotropin receptor).
